# Value based maternal and newborn care requires alignment of adequate resources with high value activities

**DOI:** 10.1186/s12884-019-2512-3

**Published:** 2019-11-21

**Authors:** Ank De Jonge, Soo Downe, Lesley Page, Declan Devane, Helena Lindgren, Joke Klinkert, Muir Gray, Anant Jani

**Affiliations:** 1 Department of Midwifery Science, AVAG, Amsterdam Public Health research institute, Amsterdam University Medical Centre, Vrije Universiteit Amsterdam, Van der Boechorststraat 7, Amsterdam, 1081 BT The Netherlands; 2 Western Sydney University, School of Nursing and Midwifery, Locked Bag 1797, Penrith, NSW 2751 Australia; 3 Research in Childbirth and Health (ReaCH Group), School of Health, College of Health and Wellbeing, University of Central Lancashire, Fylde Rd, Preston, PR1 2HE UK; 4 Visiting Professor in Midwifery King’s College London, Faculty of Nursing and Midwifery, KCL, 57 Waterloo Rd, London, SE18WA UK; 5 School of Nursing and Midwifery, National University of Ireland, University Road, Galway, H91 TK33, Ireland; 6 Department of Women’s and Children’s Health (KBH), K6, Karolinska Institute, Barnmorskeprogrammet, Retzius väg 13 A-B, plan 4, 171 77 Stockholm, Sweden; 7 EVAA Holding (Primary Care Midwives Amsterdam Amstelland), Rijtuigenhof 105, Amsterdam, 1054 NC The Netherlands; 8 Nuffield Department of Primary Care Health Sciences, Medical Science Division, Gibson Building, 1st Floor, Radcliffe Observatory Quarter, Woodstock Road, Oxford, OX2 6GG UK

**Keywords:** Value based health care, Continuity of care, Midwifery, Place of birth, Allocation of resources

## Abstract

**Background:**

Evidence based practice has been associated with better quality of care in many situations, but it has not been able to address increasing need and demand in healthcare globally and stagnant or decreasing healthcare resources. Implementation of value-based healthcare could address many important challenges in health care systems worldwide. Scaling up exemplary high value care practices offers the potential to ensure values-driven maternal and newborn care for all women and babies.

**Discussion:**

Increased use of healthcare interventions over the last century have been associated with reductions in maternal and newborn mortality and morbidity. However, over an optimum threshold, these are associated with increases in adverse effects and inappropriate use of scarce resources. The Quality Maternal and Newborn Care framework provides an example of what value based maternity care might look like. To deliver value based maternal and newborn care, a system-level shift is needed, ‘from fragmented care focused on identification and treatment of pathology for the minority to skilled care for all’.

Ideally, resources would be allocated at population and individual level to ensure care is woman-centred instead of institution/ profession centred but oftentimes, the drivers for spending resources are ‘the demands and beliefs of the acute sector’. We argue that decisions to allocate resources to high value activities, such as continuity of carer, need to be made at the macro level in the knowledge that these investments will relieve pressure on acute services while also ensuring the delivery of appropriate and high value care in the long run. To ensure that high value preventive and supportive care can be delivered, it is important that separate staff and money are allocated to, for example, models of continuity of carer to prevent shortages of resources due to rising demands of the acute services.

**Summary:**

To achieve value based maternal and newborn care, mechanisms are needed to ensure adequate resource allocation to high value maternity care activities that should be separate from the resource demands of acute maternity services. Funding arrangements should support, where wanted and needed, seamless movement of women and neonates between systems of care.

## Background

Evidence-based practice (EBP) has become the leading paradigm in healthcare since the 1990s. EBP emphasizes the need to balance research evidence, clinical experience and clients’ values and preferences in health care decisions [1]. However, ‘values’ tend to be overlooked in favour of population based norms. EBP has been associated with better quality of care in many situations, but it has not been able to address two key constraints challenging healthcare systems globally:

1. Increasing need and demand
2. Stagnant or decreasing healthcare resources

Value based health care incorporates EBP to achieve the best outcomes for patients and populations with the optimal use of resources (money, time, carbon and use of space) while, importantly, re-integrating values into the system. Three types of value are particularly emphasised [1]: 1. Personal value: the individual’s values as the basis for decision-making 2. Technical value; using resources optimally and 3. Allocative value; allocation of resources in health (or even broader: in the public sector) optimally and equitably among the population [1].

To provide high value health care, it is essential to rethink health care organisations as complex adaptive systems, and not as provider driven silos. In maternal and newborn care, this means providing care that enacts the three values above, in community, hospitals, public health and social care settings, as needed by women, babies, and families [2]. Value based health care is consistent with global movements to put women and their families (woman centred care), rather than the needs of the institution or professions, at the centre of care [2–4]. We argue that a shift is needed in maternal and newborn care from a predominant focus on acute services towards high value care provision, which includes allocating separate resources for preventive and supportive care, such as continuity of carer throughout childbirth.

## Value based maternal and newborn care

**Fig. 1:**
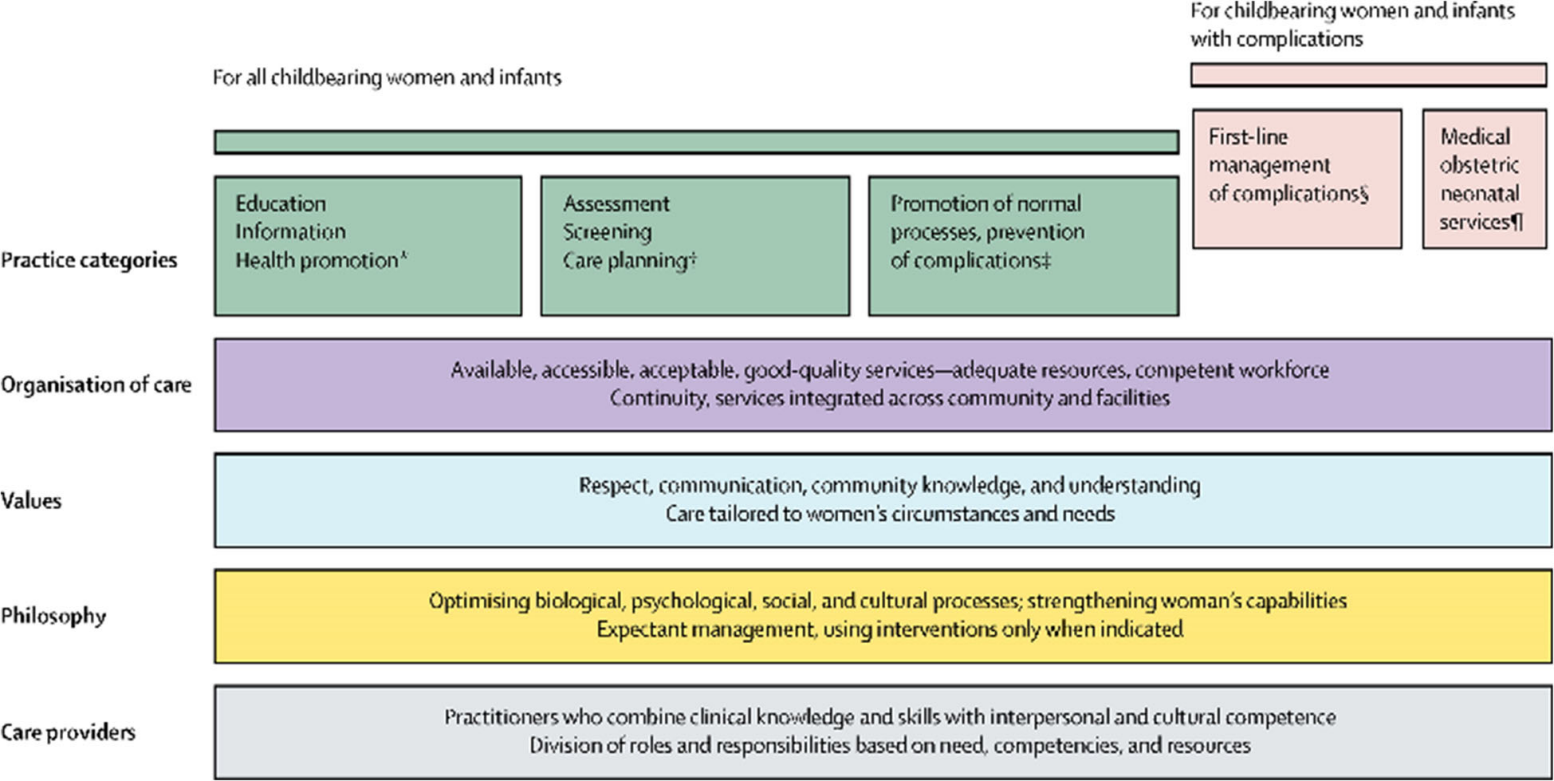
The Framework for Quality Maternal and Newborn Care (QMNC). Adjusted figure from Renfrew et al. [2] All women need midwifery care which includes management of complications and access to emergency treatments (pink areas) if required.

The “Quality Maternal and Newborn Care (QMNC) framework” defined in the Lancet provides an example of what value based maternity care might look like in practice. The QMNC framework is based on extensive reviews of qualitative and quantitative evidence of women’s views and experiences (their values), and maternal and newborn outcomes. It describes midwifery care for all women and babies, and additional specialist care for those who need it (Fig. 1) [2].

Important aspects are: a focus on prevention and supportive care to strengthen women’s capabilities and to meet their needs, promotion of normal reproductive processes and access to management of complications and emergency treatments if necessary. Midwifery care is mostly, but not exclusively, provided by midwives. Obstetricians and paediatricians mostly treat women and babies at risk of complications and provide emergency treatments. However, for women and babies in their care they provide some midwifery care, i.e. preventive and supportive care, as well.

**Fig. 2:**
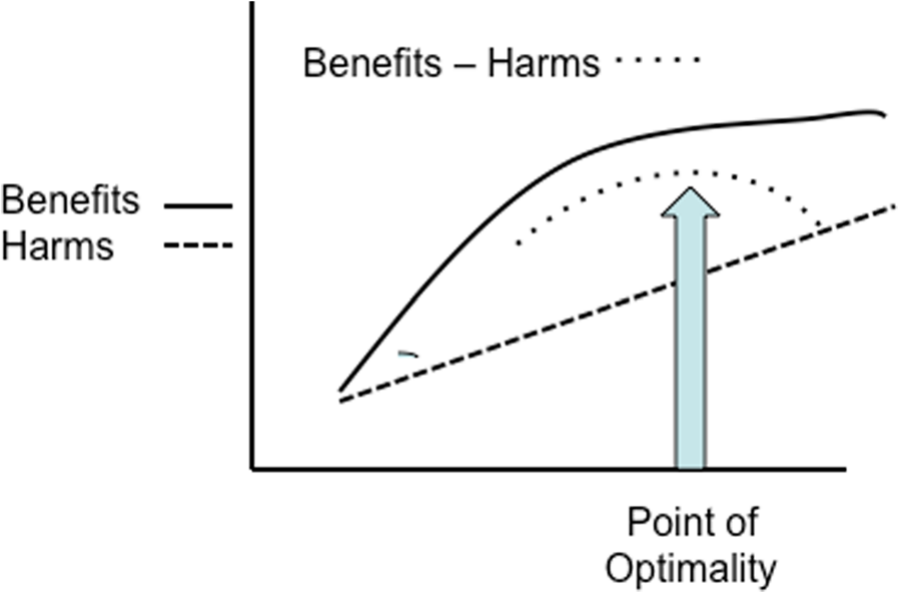
Donebedian’s graph. Relationship between investment of resources, benefits and harm [1, 6]

Increased use of medical interventions over the last century have been associated with reductions in maternal and newborn mortality and morbidity [2]. For example, a caesarean section (CS) is a life-saving operation for women and babies who need it, and improvements in neonatal intensive care have led to great improvements in the outcomes for newborns. However, over an optimum threshold, increased use of healthcare interventions in maternity care are associated with increases in adverse effects and use of scarce resources [1, 2, 5] (Fig. 2) thus limiting the potential to fund high value care provision. To deliver value based maternal and newborn care, a system-level shift is needed, ‘from fragmented care focused on identification and treatment of pathology for the minority to skilled care for all’ [2]

.

### High value maternal and newborn Care in Practice

There is substantial variation in the use of healthcare interventions and healthcare provision between different regions, healthcare organisations and care providers [2]. Variations are so large that they cannot be explained by underlying clinical characteristics or needs of women. For example, rates of CS vary from 40.5% in Latin America and 32.3% in North America to 7.3% in Africa [7]. Between European countries rates vary from 15% to over 30% [7]. Recent Lancet series have highlighted the unequal distribution of medical interventions, in particular CS, with many women receiving ‘too much too soon’ and others receiving ‘too little too late’ [8–10]. Obstetricians have called for interventions to stop the CS epidemic and to reallocate money that would become available as a result to better care which will, in turn, reduce inappropriate CS [9].

Effective midwifery care shows similar variation. Moving towards precision maternal and newborn care and promoting continuity of carer models are two possible avenues to limit unwarranted variation.

#### Precision maternal and newborn care

Effective identification of the level of care and type of care needed by each individual woman and baby will support more precise provision of appropriate care. In line with EBP and value based maternal and newborn care, personalised, clinical decisions should be based on women’s values and preferences, professionals’ clinical experience and scientific evidence. However, this type of precision maternity care is often not available. For example, planned out of hospital births have generally been associated with similar perinatal outcomes and fewer healthcare interventions compared to planned hospital births. In a meta-analysis of studies in settings where home births are well integrated in the maternity care system, the OR for intrapartum and neonatal deaths up to 28 days was 1.07 (95% CI 0.70 to 1.65) for nulliparous women and 1.08 (95% CI 0.84 to 1.38) for parous women [11]. Another meta-analysis showed similar results and lower rates of interventions but no distinction was made between nulliparous and parous women [12]. An English study of more than 60,000 women found lower rates of caesarean section (adjusted OR 0.31, 95% CI 0.23 to 0.41) among planned home versus planned hospital births and lower rates of ventouse deliveries (adj. OR 0.29, 95% CI 0.21 to 0.40), forceps deliveries (adj. OR 0.43, 95% CI 0.32 to 0.57) epidural or spinal analgesia (adj. OR 0.25, 95% CI 0.20 to 0.31) and episiotomies (adj. OR 0.33, 95% CI 0.28 to 0.39), controlled for parity and other factors [13]. Planned birth in a birth centre compared to planned hospital birth was also associated with similar neonatal outcomes and lower intervention rates [13, 14]. In England, two thirds of women would prefer to give birth at home or in maternity units, and most of these women and babies would benefit from doing so [15]. However, not all midwives are experienced and able to support these choices, and not all options for place of birth are available. The consequence is that 85% of women give birth in acute, obstetric units [15].

#### Relationship based continuity of Carer

Relationship based continuity of carer, provided by one or a small team of midwives, compared to other models of care leads to high value for women and has a high technical value [16–18]. A Cochrane systematic review of randomised controlled trials comparing midwife-led continuity of care versus other models of care showed lower rates of prematurity (RR 0.76, 95% CI 0.64 to 0.91), regional analgesia (RR 0.85, 95% CI 0.78 to 0.92), instrumental vaginal birth (RR 0.90, 95% CI 0.83 to 0.97) and fetal loss (RR 0.84, 95% CI 0.71 to 0.99). Maternal satisfaction was not measured in a uniform way, but rates were higher in most studies among women in midwife-led continuity of care models and there was a trend towards lower costs for these models.

In New Zealand, most women receive continuity of carer throughout their antenatal, intrapartum and postnatal care from their Lead Maternity Carer, regardless of risks and complications and place of birth [17]. In spite of many reported benefits of continuity of carer in Australia [19–21], only 8% of women in public hospitals currently have access to this [22]. In Canada, midwives provide continuity of carer [18]. Midwives in Canada have only recently been legalized as a profession and only 0.3 to 9.8% of all pregnant women receive care from midwives in the different provinces [18]. In England continuity of carer is provided for some women by caseload or small teams of midwives [23]. In Scotland and England, current policies aim to implement continuity of carer for all women [15, 24]. In Ireland, the provision of continuity of care is a fundamental component of its recent National Maternity Strategy, yet most women do not have access to such models of care [25].

### Barriers to implementing higher value maternal and newborn care

In 1993, the *Changing Childbirth* policy report highlighted the three Cs; women should have choice (for example in their planned place of birth), control and continuity of care [26]. Twenty-five years later, these aims have not been realised fully. To ensure more effective implementation of high value care provision, the current English *Better Births* initiative, and similar policies in Scotland and Wales, re-frame maternity care around both safety and personalised care. There are several reasons for the system inertia that followed Changing Childbirth, and other similar initiatives in the UK and in other countries, that were welcomed in principle at all levels of policy and practice, but only partly enacted in practice. These factors include a lack of strategic resource re-allocation from existing established models of care, and the associated difficulty of fundamental change in systems and cultures of care.

Financial constraints have forced healthcare services to make tough allocation decisions. Oftentimes, these decisions have limited higher value care provision, as the drivers for spending resources are ‘the demands and beliefs of the acute sector’ [26]. This is evident in maternity care in high income countries which have high rates of hospitalisation and overuse of healthcare interventions. However, in low and middle income countries too, the increase in facility births has been associated with rising CS rates and an estimated 21% of all women worldwide give birth via CS which is well above the regarded optimal level of 10–15% [8]. The priority of acute services is a focus on risk-identification and the management of complications (Fig. 1). This is appropriate where it is needed but when it is overused, it supresses focus on and resource allocation for preventive and supportive care, which reduces the overall value of maternal and newborn care. Examples of this lack of balance in maternal and newborn care provision exist in low, middle and high income countries around the world [2]. For example, developments in China, India and Brazil show that a focus on facility based and emergency care can result in better maternal and newborn survival rates but without the provision of the full spectrum of midwifery care this leads to rapidly increasing rates of (avoidable and sometimes harmful) healthcare interventions as well.

Even where birth centre and home birth options are offered, as in some places in the UK, temporary staff shortages will often result in midwives who are working in these settings being drawn back to work on acute labour wards [23]. This means that women cannot be guaranteed their choice of out-of-hospital birth. For many healthy women and babies, a birth centre birth is least likely to be associated with harm for them, so the withdrawal of midwife support in these settings also increases the risk of iatrogenic damage [15]. Rather than making birth centres the default option for women with uncomplicated pregnancies, they often remain ancillary and are closed when the service is busy.

In Sweden, an alongside birth centre (Sődra BB) ran successfully from 2007 until 2017. Among 2555 births in the birth centre, rates of medical interventions were lower and maternal and neonatal outcomes were equally good or better compared to 9382 births in standard care [27]. More women and partners were satisfied with the care in the birth centre [28]. However, when the obstetric unit needed more space, this birth centre was closed.

In Ireland, two alongside birth centres were opened in 2004. Despite evidence of their clinical, social and economic benefit, acceptance and endorsement by the Health Service Executive and a more recent National Maternity Strategy recommending the establishment of alongside birth centres [29] they remain the only two such centres in the Republic of Ireland.

In Amsterdam, the primary care midwifery organisation and both academic hospitals signed a contract with the main health insurance company in the region to build two alongside birth centres, one in each hospital. Midwives would provide continuity of care for women with low and ‘moderate’ risk indications, such as request for epidural anaesthesia. However, professionals were told by the hospital boards: ‘ *because secondary and tertiary maternity care are our core business, we have been forced to give priority to building processes linked to this care’*. The creation of the birth centres has been postponed while increasingly labouring women cannot be admitted to obstetric units because they are too busy, leading to unsafe and dissatisfying situations.

### Allocating resources to higher value care

Recently, a Blueprint was published for ‘Advancing High-Value Maternity Care Through Physiological Childbearing’ in the United States which recommends that payments are aligned with high-value maternity care [5]. Similarly, an English policy document recommends services to ‘incentivise the delivery of high quality of care for all women’ [15].

Demands will inevitably always outweigh available resources in the health sector. At the provider level, for professionals on the working floor it makes sense to prioritise acute services. For example, when managing a busy labour ward, making sure that an epidural or CS can be performed in a timely and safe manner is more important than providing personalised, continuity of care. But at the payer level, prioritising preventive care that is woman-centred will provide higher value for the population as a whole.

Ideally, resources would be allocated at a population and individual level to ensure care is woman-centred instead of institution/ profession centred but oftentimes, imbalances favouring the acute services have limited access to higher value provision for women. We argue that decisions to allocate resources to higher value activities need to be made at the macro level in the knowledge that these investments will also relieve pressure on the acute services in the long run [15]. To ensure that high value preventive and supportive care are available and can be delivered, it is important that resources are allocated separately for these activities; staff and money allocated to, for example, birth centres and continuity of carer models need to be separated from resources for acute services to prevent re-allocation of resources from non-acute to acute services because of rising demands of acute services.

Allocation of separate resources for these high value activities underscores their importance. It gives the message to professionals and the public that access to and choice of continuity of carer throughout childbirth, for example, is as important for all women as acute services are for women who need it. In fact, the provision of continuity of carer will reduce the need for acute services (for example for preterm labour or instrumental birth) and therefore facilitate the availability of these services when required. It also enables the continuation of continuity of carer when additional specialist care is needed.

## Conclusions

To implement value based maternal and newborn care, women’s values should be at the heart of maternity care. However, good intentions are not sufficient to deliver results. To overcome imbalances favouring acute services, resources should be allocated separately to high value activities, such as continuity of carer models and out-of-hospital services. If all care is funded from one budget, earlier experiences show that high technological care will often be prioritised over personal continuity of carer even for women for whom the latter is of higher value. It is important to evaluate how maternity services in a region can achieve the best outcomes for the optimal use of resources. To achieve value based maternal and newborn care, systems are needed to ensure adequate resource allocation to high value maternity care activities that are separate from the resources of acute maternity services. Funding arrangements should support, where wanted and needed, seamless movement of women and neonates between systems of care.

## Data Availability

Not applicable.

## Abbreviations

CS: Caesarean section(s)
EBP: Evidence based practice
QMNC framework: Quality Maternal and Newborn Care Framework
